# Fully personalized modelling of Duchenne Muscular Dystrophy ambulation

**DOI:** 10.1098/rsta.2024.0218

**Published:** 2025-04-02

**Authors:** Victor Applebaum, Evan Baker, Thomas Kim, Georgia Stimpson, Peter Challenor, Kyle Carlton Abesser Wedgwood, Matthew Anderson, Ian Bamsey, Giovanni Baranello, Adnan Manzur, Francesco Muntoni, Krasimira Tsaneva-Atanasova

**Affiliations:** ^1^ Department of Mathematics and Statistics and EPSRC Hub for Quantitative Modelling in Healthcare, University of Exeter, Exeter, UK; ^2^ Certus Technology Associates Ltd, Exeter, UK; ^3^ Dubowitz Neuromuscular Centre, NIHR Great Ormond Street Hospital Biomedical Research Centre, Great Ormond Street Institute of Child Health, University College London, Great Ormond Street Hospital Trust, London, UK

**Keywords:** Duchenne Muscular Dystrophy, personalized medicine, dynamic linear model, Gaussian process, synthetic data, patient trajectories

## Abstract

Duchenne Muscular Dystrophy is a progressive neuromuscular disorder characterized by the gradual weakening and deterioration of muscles, leading to loss of ambulation in affected individuals. This decline in mobility can be effectively assessed using the North Star Ambulatory Assessment (NSAA) scores, along with measures such as the 10-m walk time and the time taken to rise from the floor. We propose a dynamic linear model to predict the trajectories of these clinical outcomes, with a primary focus on NSAA scores. Our model aims to assist clinicians in forecasting the progression of the disease, thereby enabling more informed and personalized treatment plans for their patients. We also evaluate the effectiveness of our models in generating synthetic NSAA score datasets. We assess the performance of our modelling approach and compare the results with those of a previous study. We show that the most robust model demonstrates narrower prediction intervals and improved quantile coverage, indicating superior predictive accuracy and reliability.

This article is part of the theme issue ‘Uncertainty quantification for healthcare and biological systems (Part 2)’.

## 1. Introduction

Duchenne Muscular Dystrophy (DMD) is an X-chromosome linked neuro-muscular degenerative disease that affects approximately 19.8 males born per 1 00 000 globally [1]. The disorder is the result of a mutation that prevents the production of muscle-specific dystrophin, a protein that stabilizes the structure of cell membranes [2]. As a result, individuals with DMD experience a deterioration of muscular abilities and strength, typically losing ambulation by the teens. The disease is associated with early-life mortality as a result of breathing difficulties, with median age of survival at around 28 years [3].

The course of a patient’s health condition over time is referred to as their patient trajectory. This includes changes in symptoms, functional abilities and overall well‐being, as well as the impact of medical interventions. The ability to predict patient trajectories offers clinicians valuable insights into future health outcomes and the effects of interventions, enabling the development of personalized treatment plans that enhance patient care. In the case of DMD, consideration of patient trajectories would be particularly beneficial since there currently is no cure for the disorder and, although a number of therapeutics have achieved some success in clinical trials [4], many are associated with adverse effects. For example, corticosteroids, one of the most common therapeutic interventions for DMD, improves motor function but also causes a number of adverse effects, such as behavioural changes, difficulty sleeping and an increased risk of bone fractures [5]. The careful selection of which corticosteroid type, regime and dose should be administered is therefore of significant importance to patients’ care, and is informed by consideration of the likely trajectory of the patient. Similarly, other interventions, such as prescription of exercise regimes, may change in line with the patient trajectory.

Mathematical models represented by dynamical systems present a powerful way to investigate patient trajectories. By fitting these models to trajectory data, parameter estimation can be performed, enabling interpolation and extrapolation for data prediction. Moreover, such models allow for analysis of variable interactions, thus enabling understanding of underlying system mechanisms.

Models can also be used to simulate trajectories of non-existent patients, i.e. synthetic data. The utility of synthetic clinical data focusing on data privacy and dataset size has been discussed extensively in Giuffrè [6] and Hahn [7]: medical datasets often contain sensitive information and pose a risk of patient re-identification. This creates barriers for access and hence limits research output. Synthetic datasets address this issue by containing no real patient information, thereby reducing regulatory and ethical concerns. Additionally, small dataset sizes often hinder the application of advanced analytical techniques (e.g. machine learning and AI), making synthetic data a valuable supplement in such cases.

In this paper, we propose a model for personalized DMD patient trajectories. Such a model gives researchers a tool to assist with determining the effectiveness of interventions, and clinicians a tool to make more informed decisions on when and if to deliver these interventions. We test the model’s ability to make these predictions accurately and precisely, and its ability to generate high quality synthetic patient data, in order to ensure that the synthetic trajectories reflect the properties in the real data. There have been a variety of attempts at investigating and modelling DMD progression in the past, often involving subgrouping/clustering approaches [8,9]. While this approach can be effective, intuitive and easily explainable, little individual variability is captured. If a patient does not fit well into any of the trajectory classes, they would still have to be allocated to a class (likely according to some similarity/distance measure). This could inadvertently affect the accuracy of any predictions for such a patient. Similarly, a patient might fit well into one class initially, but be better described by another class at a later time. As such, these clustering methods are well-suited for stratification, but they can struggle with more precise and personalized modelling goals. Other modelling approaches involve the construction of models with random effects terms. This accounts for parameter variation in individuals as part of a population, reducing the impact of the issues with clustering discussed above. Previous studies utilizing this technique have modelled changes in biomarker indicators and leg fat fraction [10]; progression in DMD patients’ 6-min walk times [11,12]; as well as progression of North Star Ambulatory Assessment (NSAA) scores [13].

In the studies discussed in the previous section, little focus is placed on individual-level analysis. This approach holds advantages as it allows for more precise predictions and treatment programme to be made for each patient, improving their quality of care. In this paper, personalized models for each patient are constructed using extensive use of hierarchical modelling. This technique allows information to be drawn from the trajectories in the population and update this according to data measurements for each individual. This is particularly useful for working with datasets where there are relatively few data points available for each individual, as power can be drawn from the population-level data. We thus make predictions and evaluate the models at the individual level, representing an advancement over the current state of DMD progression modelling, which has predominantly focused on group-level and population-level modelling. Of the studies we are aware of Hibma [13] comes closest to creating an individual-level model for NSAA scores, accounting for individual variability using random effects. We therefore additionally extend the model presented in that paper to be fully individualized through the same hierarchical approach and compare it with our own.

Our modelling framework is based on a dynamic linear model (DLM). An advantage of our model is that while it has enough complexity to capture the various relationships, it is also simple enough to be explainable and easily generalized for use in modelling other diseases.

We also explore modelling multiple clinical metrics at once using a covariate structure. By doing so, we are able to extract more information about parameters for each individual during the model fitting stage, decreasing uncertainty around the parameter estimates. Additionally, because our approach is based on DLMs, we separate observational noise from structural variability, allowing more complex trajectories than our parametrization would usually allow, and we can easily accommodate missing observations. Our approach also uses a robust description of uncertainty, employing Bayesian prediction intervals (PIs). This framework allows uncertainty in predicted outcomes to be seen by clinicians, allowing more informed decisions on interventions to be made, and also enables observations of where this uncertainty comes from to be made, thus enhancing interpretability of our predictions.

We focus on the NorthStar Ambulatory Assessment (NSAA) score as a measure of DMD patients’ ambulatory abilities, which is a validated measure of the patients overall condition and has been used in many clinical trials for DMD interventions [14–18]. The NSAA is a 17-item assessment of motor function, which includes standing, running and hopping. Each item can be scored as a 0 (no ability), 1 (able with compensation) and 2 (able with no compensation), for a total score of 34. The dataset contains two other measures that we also model: patients’ 10-m walk times and time to rise from floor (RFF) time—i.e. time to rise from a supine lying-down position to standing.

An outline of our paper is as follows: in §2, we describe the dataset we use and define and explain the proposed models. In §3, we then explain how we assess the quality of the predictions the models make, before presenting the results in §4. We then describe our methodology for generating robust synthetic data in §5 and present the results in §6. We discuss the models and results further in §7.

## 2. Data and our modelling procedure

Here, we provide details of the clinical data we model and describe our proposed methodology.

### (a) Data and software

The dataset we use is from the NorthStar Clinical Network Database. It contains values for three dependent variables: NSAA score, walk time and RFF time. We have an input space of time since birth in ${\mathbb{R}}_{+}$ , as well as outputs of NSAA score in {0,1,...,34} , walk times in ${\mathbb{R}}_{+}$ and RFF times in ${\mathbb{R}}_{+}$ .

The dataset contains scores from 1018 DMD patients, including 4867 walk time entries, 4636 RFF time entries and 5989 entries for NSAA score.

In the process of developing our data-driven modelling framework, we split the entire dataset as follows: 294 (approximately 30%) randomly sampled patients are set aside as a validation dataset. The remaining 724 (approximately 70%) individuals are used as training data for the model’s population-level parameters.

Before analysis, some adjustments and reparameterizations were carried out on the dataset. Firstly, time points were grouped by three-month periods, rather than specific dates, meaning that each data entry has a time value in the set {1,2,...,69} , which represents ages from 3 up to 20.25 years. This time‐step choice enables the model to roughly follow clinical appointments, which aim to follow a regular six-month schedule. Moreover, the time step is small enough to accurately determine whether each data point falls early or late within a six-month window. The length also reduces the computational intractability that would arise from choosing too small a time step, while preserving numerical stability.

When developing our models (as opposed to the Hibma model, which will be discussed later), reparameterizations were chosen to ensure the outcome predictions for NSAA score, walk time and RFF time were constrained to {0,1,...,34} , ${\mathbb{R}}_{+}$ and ${\mathbb{R}}_{+}$ , respectively. NSAA scores were transformed via logit((NSAA+0.5)/35) and then normalized (mean value subtracted, then divided by the standard deviation). Similarly, walk time and RFF time were transformed using a softplus function and normalized. The additional normalization procedure was carried out in order to keep the data on a consistent scale between individuals and to improve numerical stability. Model development was then carried out in this transformed space. During analysis, predictions were converted back using the inverse of these transformations.

All computations were conducted in R version 4.2.2 [19]. Model development was performed based on Bayesian inference with Markov Chain Monte Carlo (MCMC) using the *NIMBLE* package [20,21]. MCMC outputs were checked visually for a constant mean and variability around that mean, as well as for the mixing of two chains.

### (b) The general model

The general structure of the models takes the following form, over time points up to 69 :


(2.1) $$\begin{array}{lcr} & y_{i,t}^{\left(k\right)}∼D\left(\theta_{i,t}^{\left(k\right)}-\beta_{i}^{\left(k\right)}\phi_{i,t}^{*}\right), & \end{array}$$

where D represents either a ‘random walk+noise’ structure or a Gaussian process (GP), as will be discussed in §2c. Subscript i indexes individuals, t time and k the outcome of interest (NSAA score, walk time, RFF time). We use $\theta_{i,t}^{\left(k\right)}$ as a latent state representing the improvement in ambulatory abilities typically observed in a healthy child as they mature. This is constructed using a term, $\delta_{i}^{\left(k\right)}>0$ , which improves the condition of the patient linearly, and $\alpha_{i}^{\left(k\right)}\in \left(0,1\right)$ , which flattens this as time increases and the individual reaches maturity:


(2.2) $$\begin{array}{lcr} & \theta_{i,t}^{\left(k\right)}=\alpha_{i}^{\left(k\right)}\theta_{i,t-1}^{\left(k\right)}+\delta_{i}^{\left(k\right)}. & \end{array}$$

The second latent state, $\phi_{i,t}^{*}$ represents the progression of the disease, and is scaled for each outcome by $\beta_{i}^{\left(k\right)}$ . We model $\phi_{i,t}^{*}$ as a softplus of another latent state, in order to constrain it as positive:


(2.3) $$\phi_{i,t}^{*}=\text{softplus}(\phi_{i,t})=\log\left(1+\exp\left(\phi_{i,t}\right)\right).$$

In turn, $\phi_{i,t}$ is modelled to increase linearly according to a parameter, $\Delta_{i}$ , as shown,


(2.4) $$\phi_{i,t}=\phi_{i,t-1}+\Delta_{i}-\Gamma_{i}x_{i,t}.$$

Here, an additional set of terms for treatment, $\Gamma_{i}x_{i,t}$ are sometimes added to equation (2.4). This will be discussed in §2c.

Ordinarily, we might estimate for the initial value $\phi_{i,0}$ . However, due to the use of the softmax transform, these become negative numbers with little meaning. Instead, we introduce $t_{i,mat}$ , representing the times at which the impact of the disease becomes relevant, i.e. when ϕ=0 . The initial value can then be found using $\phi_{i,0}=-t_{i,mat}\Delta_{i}$ . Similarly, we find the values of δ through alternative means. In this case, we find the initial value of the healthy state $\theta_{i,0}^{k}$ , and $\theta_{i,\text{mat}}^{k}$ , which represents the value of the healthy state after the patient has reached full maturity. For fitting, we assume this age to be 20 years, representing time step $t_{mat}=68$ , although flexibility in our model design means that $\theta_{i,t}^{k}$ is not forced to reach its maximum at this time. As we have a geometric series:


(2.5) $$\begin{array}{lcr} & \theta_{i,mat}={\alpha_{i}}^{t_{mat}}\theta_{i,0}+\sum_{t=0}^{t_{mat}}\delta_{i}{\alpha_{i}}^{t}={\alpha_{i}}^{t_{mat}}\theta_{i,0}+\delta_{i}\frac{1-{\alpha_{i}}^{t_{mat}}}{1-\alpha_{i}}, & \end{array}$$

with k sub-scripting omitted, $\delta_{i}^{\left(k\right)}$ can then be calculated using the analytical formula:


(2.6) $$\delta_{i}^{\left(k\right)}=\left(\theta_{i,\text{mat}}^{k}-\alpha_{i}^{(k{)}^{t_{mat}}}\theta_{i,0}^{k}\right)\frac{1-\alpha_{i}^{\left(k\right)}}{1-\alpha_{i}^{(k{)}^{t_{mat}}}}.$$

By subtracting the disease state, $\beta_{i}^{\left(k\right)}\phi_{i,t}^{*}$ , from the healthy state, $\theta_{i,t}^{\left(k\right)}$ , we hence attain a description of the state of the patient.

All parameters defined above are modelled hierarchically over the individuals using a normal distribution, with weak priors on the mean and standard deviation terms. Details of these can be found in electronic supplementary material A.

### (c) Model variations

With the purpose of creating a model able to produce the most accurate and narrowest predictions, as well as the most similar synthetic datasets, we investigate the advantages and disadvantages of three modelling choices: how to assess model discrepancy, whether it is useful to covary parameters using co-predictors and whether there is value to modelling treatments. Below we explain these variations in detail and use them to propose eight variations of the model that can be compared against one another. We also incorporate an adaptation of a previous model from Hibma [13] into our framework for comparison.

#### (i) Gaussian processes

As the real-world dataset we are using is characterized by a significant level of variability (heterogeneity) along with low numbers of data points per individual, we can expect high prediction uncertainty: the difference between the model prediction and the actual measured values. Therefore, a vital aspect of our model is how it quantifies this uncertainty.

One of the modelling approaches for quantifying this uncertainty, which we explore in this paper, is the GP. In GPs, the distribution of the points in the space can then be modelled using a multidimensional normal distribution, N .

Briefly, as described in [22, pp. 146−151] and [23], GPs function as follows: firstly, a covariance kernel, K , is chosen for the model. This must be a positive semi-definite function. A typical choice, due to its smoothness, is the squared exponential kernel,


(2.7) $$\begin{array}{lcr} & K_{\rho ,\lambda }(x_{1},x_{2})=\rho^{2}exp\left(-{\left(\frac{x_{1}-x_{2}}{\lambda }\right)}^{2}\right), & \end{array}$$

which has hyperparameters ρ and λ . The prior distribution of curves is then modelled as


(2.8) $$\begin{array}{lcr} & y_{T}∼N(\mu (T),K(T,T)), & \end{array}$$

where T is the set of time points, K(⋅,⋅) is the N×N matrix generated by the kernel function and T is our time domain, {1,2,...,69} . When a dataset, with domain X and codomain Y has been investigated, a posterior can be calculated. The analytical posterior distribution is as follows [24, pp. 116−117]:


(2.9) $$y_{T}|X,Y∼N(\bar{\mu }(T),\bar{K}(T))$$

with mean


(2.10) $$\bar{\mu }(T)=\mu (T)+K(T,X)\Sigma (X,X{)}^{-1}(Y-\mu (X))$$

and variance


(2.11) $$\bar{K}(T)=K(T,T)-K(T,X)\Sigma (X,X{)}^{-1}K(T,X{)}^{⊤},$$

where ⊤ represents a transpose.

Here, Σ is defined as follows,


(2.12) $$\Sigma (X,X)=K(X,X)+\tau_{obs}^{2}I,$$

where I is an identity matrix. The term includes a positive number representing white noise, $\tau_{obs}^{2}$ , which can represent uncorrelated noise from aleatoric sources. This increases uncertainty around known design points (known points (X,Y) from the dataset), allowing the data to be distributed normally around the posterior mean line.

When using GPs in our models, we denote the white noise term as $\tau_{obs,i}^{\left(k\right)}$ , which can be represented as a vector added to the estimate. We can then write equation (2.1) as


(2.13) $$\begin{array}{lcr} & y_{i,T}^{\left(k\right)}∼GP\left(\theta_{i,T}^{\left(k\right)}-\beta_{i}^{\left(k\right)}\phi_{i,T}^{*},K_{\rho_{i}^{\left(k\right)},\lambda_{i}^{\left(k\right)}}(T,T)\right)+\left[\begin{array}{c} \tau_{obs,i}^{\left(k\right)}\\ \vdots \;\\ \tau_{obs,i}^{\left(k\right)} \end{array}\right]. & \end{array}$$

Here, GP is equivalent to N . X and Y in equations (2.9)–(2.11) then represent the times and scores for individual i for outcome of interest k . The GP hyperparameters are modelled from a hierarchical structure similar to that of the model parameters.

#### (ii) Random walk structure

The random walk approach takes a simpler view than that of the GP, adding a random walk term, $\epsilon_{t}$ at each time step, t , representing a deviation not represented in the model, but still part of the overall trajectory, in addition to an aleatoric noise term, $\tau_{obs}$ . We write:


(2.14) $$\begin{array}{lcr} & y_{t}∼N(\mu_{t}+\epsilon_{t},\tau_{obs}), & \end{array}$$

with


(2.15) $$\begin{array}{lcr} & \epsilon_{t}∼N(\epsilon_{t-1},\tau_{innov}), & \end{array}$$

where $\tau_{innov}$ represents the standard deviation of the random walks step sizes.

In random walk models, equation (2.1) becomes


(2.16) $$\begin{array}{lcr} & y_{i,t}^{\left(k\right)}∼N\left(\theta_{i,t}^{\left(k\right)}-\beta_{i}^{\left(k\right)}\phi_{i,t}^{*}+\epsilon_{i,t}^{\left(k\right)},\tau_{obs,i}^{\left(k\right)}\right), & \end{array}$$

with


(2.17) $$\begin{array}{lcr} & \epsilon_{i,t}^{\left(k\right)}∼N\left(\epsilon_{i,t-1}^{\left(k\right)},\tau_{innov,i}^{\left(k\right)}\right). & \end{array}$$

As in other parts of the model, we use a hierarchical prior system on $\tau_{obs,i}$ , $\tau_{innov,i}$ , $\epsilon_{i,0}$ .

#### (iii) Covariate usage

We have three dependent variables: NSAA score, walk time and RFF time. When modelling each of these, there is the opportunity to use the other two variables as covariates to estimate some of the model parameters. We now consider whether this would be effective and how to do this.

For individuals with low numbers of data points, it can be useful to use covariates to extract more information about the trajectory. However, this introduces more parameters, which can result in issues with overparameterization, such as non-identifiability and overfitting.

We investigate using covariate terms on the α , β , $t_{0}$ and $\theta_{\text{mat}}$ parameters. This means that rather than placing hierarchies on the mean and standard deviation terms of a normal distribution for each clinical outcome (dependent variable), we instead model the outcomes together in a multivariate normal distribution, placing hierarchies on the mean vector and covariance matrices in those:


(2.18) $$\begin{array}{lrlr} & \alpha_{i}^{\left(k\right)} & ∼N(\mu_{\alpha^{\left(k\right)}},\sigma_{\alpha^{\left(k\right)}}\Sigma_{\alpha^{\left(k\right)}}), & \end{array}$$

(2.19) $$\begin{array}{lrlr} & \beta_{i}^{\left(k\right)} & ∼N(\mu_{\beta^{\left(k\right)}},\sigma_{\beta^{\left(k\right)}}\Sigma_{\beta^{\left(k\right)}}), & \end{array}$$

(2.20) $$\theta_{i,0}^{\left(k\right)}∼N(\mu_{\theta 0},\sigma_{\theta 0}{\text{Corr}}_{\theta 0}),$$

(2.21) $$t_{i,\text{mat}}^{\left(k\right)}∼N(\mu_{t\text{mat}},\sigma_{t\text{mat}}{\text{Corr}}_{t\text{mat}}),$$

where Σ and Corr are positive definite symmetric matrices with unit diagonals, and σ are scalar multipliers.

#### (iv) Treatments

Including when and how a patient is being treated could be a useful part of the model. This is because it allows us to extract population-level treatment parameters to see how effective the treatments are, enabling clinicians to determine how an outcome would change if the treatments were given. However, this strategy poses some challenges in practice. Patients who are not on steroids are not necessarily a control for patients who are, and this potentially creates non-identifiability issues in the model. This can, for example, underestimate treatment effects or even falsely suggest that the treatments negatively affect ambulation. Regardless, the shapes of the trajectories in those who are taking the treatments and those who are not may be different, and this is an important aspect of our investigation.

When we choose to model treatments, we do so in the deterioration part of the model, equation (2.4). We have the treatment’s effect as a positive vector $\Gamma_{i}$ , where different elements represent the effects of different treatments on that individual. This is multiplied by another vector, $Q_{i,t}$ , in which each element contains a 1 or 0 , indicating if they are taking or not that treatment at time t . Equation (2.4) is thus as described when treatments are being included, and as follows when not:


(2.22) $$\phi_{i,t}=\phi_{i,t-1}+\Delta_{i}.$$

The treatment effect $\Gamma_{i}$ is modelled hierarchically, with a softplus function used to ensure the treatment term is always positive. For each treatment p , we have:


(2.23) $$\begin{array}{ll} {\text{softplus}}^{-1}(\Gamma_{i,p})=log(exp(\Gamma_{i,p})-1) & ∼N(\mu_{\Gamma_{p}},\sigma_{\Gamma_{p}}^{2}). \end{array}$$

#### (v) Hibma model

We aim to compare our models with a previously proposed model: the base-NSAA model presented in Hibma [13], which exclusively works on NSAA scores (i.e. not walk times or RFF times). It should be noted that this model appears to be primarily designed with stratification and simulation, rather than future score prediction, in mind. We will refer to this as the Hibma model. It takes the form of a modified indirect response model, and is simpler than our models, with fewer parameters. We adapt it into our framework as follows:


(2.24) $$\begin{array}{lcr} & y_{i,t}^{\left(1\right)}∼N(y_{i,t-1}^{\left(1\right)}+\phi_{i}-t\xi_{i}y_{i,t-1}^{\left(1\right)}+\epsilon_{i,t},\tau_{obs,i}). & \end{array}$$

Here, $\phi_{i}$ represents the effect of a production rate constant, and $\xi_{i}$ represents the effect of the dissipation rate constant. The names of the constants are standard in indirect response models, and do not necessarily reflect any actual production or dissipation that might be occurring, however, they control the increase and decrease rates in the scores we predict. We place hierarchical priors on these parameters. Note that we also add the random walks, $\epsilon_{i,t}$ , defined the same way as in §2c(ii), which is not included in the original study.

The model is fitted in the non-reparameterized space, i.e. without transforming with logit((NSAA+0.5)/35) and without normalizing, as this was not performed in the Hibma paper when the model was designed. While this means that predictions can increase above 34 and below 0 , we enforce these restrictions by taking the maximum of 0 and the estimated value as the true prediction, and similarly, the minimum of 34 and the estimated value for the upper bound.

#### (vi) Defining the models

We create eight models, labelled A–H, with the purpose of having one model for each combination of the above discussed variations. The Hibma model is additionally given the label I. table 1 shows which model has which variations and full statements of all models can be found in electronic supplementary material A.

**Table 1.** Table of which model contains which variations. In the covariates and treatments rows, these refer to whether covariates and treatments are included in the model. In the discrepancy row, RW refers to the random walk + noise structure, while GP refers to a Gaussian process. Model I represents our adaptation of the model presented in [13].

model	A	B	C	D	E	F	G	H	I
discrepancy	RW	RW	RW	RW	GP	GP	GP	GP	RW
covariates	yes	yes	no	no	yes	yes	no	no	NA
treatments	yes	no	yes	no	yes	no	yes	no	yes

## 3. Methodology for evaluation of prediction quality

In order to create a personalized model calibration, we select the first 20–80% (rounded down) data points uniformly for each individual to be the prediction points for that individual. For example, if we observe an individual with 9 points and sample 25% , this will represent the first 2.25 points, which would be rounded down to the first 2 . These early points are used to calibrate the model for the individual as detailed below. We use the remaining data points of each individual for validation.

The MCMC procedure generates posterior samples for the population-level parameters based on the training data. We approximate these with a multivariate normal distribution, which is able to capture covariances within the posterior. Those parameters, which are truncated at zero, are reparameterized using a log function, to keep their shape and avoid the distribution including values below zero. Visualizations of this approximation can be seen in electronic supplementary material I. We then obtain posterior samples for each new individual, given the individuals’ prediction data points, by rerunning the MCMC procedure effectively as a fixed-effects model with the multivariate distribution prior.

A posterior predictive distribution can then be generated to calculate PIs. Ideally, for a well calibrated model, an w% PI should contain w% of the validation points. We verify whether this is the case visually using QQ plots by plotting PI against the proportion of validation points these intervals contain. Additionally, we use the normalized sum of the absolute differences (SAD) between the two values to devise a score for the prediction performance, where a lower score represents a better performing model:


(3.1) $$\text{SAD}=\sum_{i\in I}|i-W_{i}|,$$

where I={0.01,0.02,...,0.99} and $W_{i}$ is the proportion of validation points within the i PI:


(3.2) $$W_{i}=\frac{\#\text{validation points}\in \text{PI}}{\#\text{validation points}}.$$

When models perform similarly in terms of calibration, we favour the model that can provide narrower PIs. To quantify this, we find the average width of the 70% and 90% PIs over the time period for each individual, and these can be then compared.

In order to describe how the models perform when different amounts of data are available, we group the individuals by number of prediction points (that is, the first 20%–80% of points for the validation individual which we allow the model to see during the validation step). We create four classes of individuals in the validation dataset for this purpose: those with 0 prediction points, 1−3 prediction points, 4−6 prediction points and 7+ prediction points. For NSAA scores, these contained 44, 161, 67 and 22 individuals, respectively. The case of 0 prediction points represents those individuals for which the percentage selected for number of prediction points would represent under 1 point, and thus was rounded down to zero, meaning no information was known and the prediction was made based on population-level information alone.

In addition, we consider how narrow a PI must be in order to be useful, and how much narrower than a PI from another model it must be to be sufficiently more useful than another model to draw conclusions from. One measure that can be used for this is the minimum clinically important difference (MCID). MCIDs are discussed in depth in Copay 2007 [25], and represent the smallest change in value that would be considered clinically meaningful. In Gupta 2023 [26], an investigation into MCIDs in NSAA scores for patients with DMD is conducted using three different approaches. One of these is the anchor-based approach, which is an estimate of the decrease in NSAA score which would result in decrease in 6-min walk distance of 30 m, using data from the iMDEX natural history study. We consider this approach particularly valuable because it links changes in NSAA scores to a tangible decline in patient abilities. The study determined the MCID for this method to be a change in NSAA score of 3.5. We therefore will be presenting NSAA score width results in terms of MCID width ,


(3.3) $$\text{MCID width}=\frac{\text{NSAA width}}{3.5},$$

as well as in terms of the NSAA score itself. If the MCID width of predictions given by a model is one or less, then this would represent sufficient precision that a further decrease in the width would have limited utility. It thus could be thought of as representing a target level of certainty for the models. By viewing the widths of the PI in terms of the MCID, we can give an idea of how close the model is to this target. More importantly, when comparing models, a decrease in this width of one or more, from one model to another, would represent an improvement in model certainty that would be clinically relevant and useful.

## 4. Models E and F outperformed the other models

In this section, we investigate how effective the models are at making predictions about the patients’ trajectories, considering each outcome type separately. Firstly we examine NSAA scores, providing full results. We then state the key results from the walk time and RFF time predictions, but provide the full results and tables in electronic supplementary materials G and F.

### (a) NSAA score prediction

**Figure 1.**
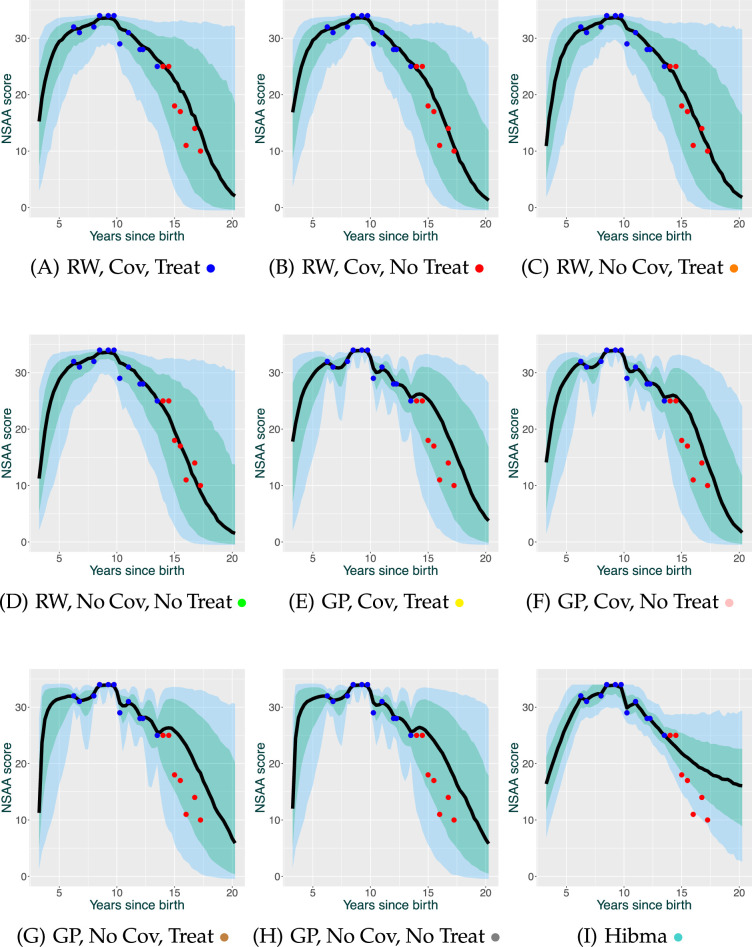
Predictions for NSAA score for a single patient. Blue shows training points, while red points are validation points. The black line, green space and blue space show the median prediction, 70% PIs and 95% PIs. Figure labels correspond to their model label, as defined in table 1. Colour keys for comparison with the QQ plots are also provided.

Firstly, we analyse the performance of the model at NSAA score predictions. An example of predictions made by the models for a patient can be seen in figure 1. Further examples can be seen in electronic supplementary material B.

On an initial inspection, the models appear to generate reasonable trajectory shapes, in particular, beginning with an increase that peaks and then falls to zero.table 2

**Table 2.** Table of SAD scores for NSAA predictions. A lower score represents better model calibration, as indicated in theright-most column.

Model	Description	SAD	Rank
A	RW, Cov, Treat	0.66	2
B	RW, Cov, No Treat	0.64	1
C	RW, No Cov, Treat	2.26	3
D	RW, No Cov, No Treat	2.42	5
E	GP, Cov, Treat	2.58	6
F	GP, Cov, No Treat	2.39	4
G	GP, No Cov, Treat	3.13	7
H	GP, No Cov, No Treat	3.68	8
I	Hibma	11.94	9

**Figure 2.**
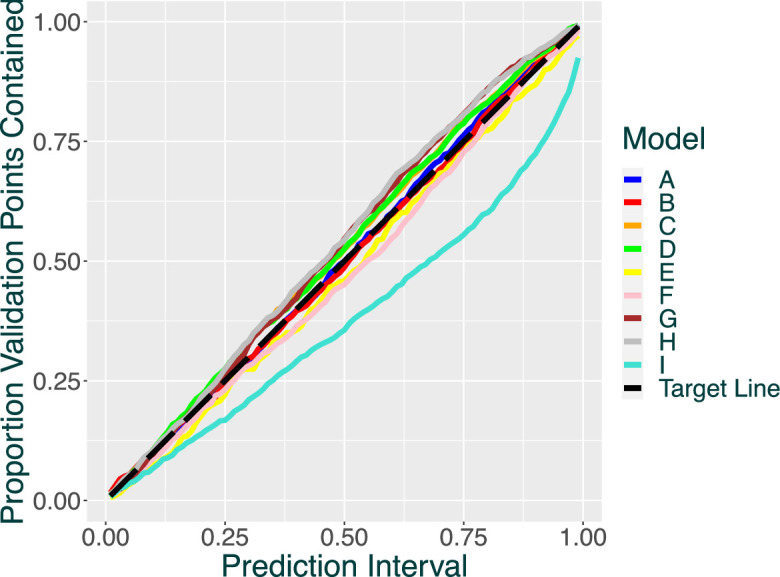
QQ plots for NSAA predictions. Plots closer to the dashed black target line have better quantile coverage, meaning stronger calibration and predictive accuracy.

We now quantify the performance of the models. The quality of the calibration can be deduced from figure 2, which contains QQ plots, and table 2, which contains the SAD scores.

**Table 3.** NSAA score PI widths for 70% and 95% PIs, displayed in terms of the scores and MCIDs in square brackets. For each width and training point group, we also highlight the best performing model in blue and the worst in orange.

model label	model description	*width with 0 training points*		width with 1–3 training points		width with 4–6 training points		width with 8+ training points	
		70%	95%	70%	95%	70%	95%	70%	95%
A	RW, Cov, Treat	15.29 [4.62]	29.41 [7.96]	11.66 [3.33]	22.82 [6.52]	10.99 [3.14]	21.07 [6.02]	11.29 [3.22]	19.80 [5.66]
B	RW, Cov, No Treat	16.88 [4.37]	27.84 [8.43]	**10.49 [3.00]**	**20.51** **[5.86]**	10.14 [2.90]	19.47 [5.56]	9.79 [2.80]	16.63 [5.21]
C	RW, No Cov, Treat	16.74 [4.78]	29.96 [8.56]	11.92 [3.41]	23.68 [6.77]	11.22 [3.21]	21.69 [6.20]	11.60 [3.31]	20.20 [5.77]
D	RW, No Cov, No Treat	17.19 [4.78]	29.90 [8.51]	11.77 [3.36]	23.41 [6.69]	10.86 [3.10]	21.22 [6.06]	10.89 [3.11]	19.71 [5.63]
E	GP, Cov, Treat	**14.29 [4.08]**	25.96 [7.42]	11.16 [3.19]	21.88 [6.25]	9.54 [2.73]	18.64 [5.89]	8.82 [2.52]	16.00 [4.57]
F	GP, Cov, No Treat	14.75 [4.21]	**25.75 [7.36]**	10.97 [3.14]	20.74 [5.92]	**9.18 [2.62]**	**17.68 [5.05]**	**8.19 [2.34]**	**15.38 [4.21]**
G	GP, No Cov, Treat	22.27 [6.36]	33.81 [9.66]	15.40 [4.40]	27.74 [7.82]	11.29 [3.22]	21.97 [6.28]	10.60 [3.03]	18.85 [5.39]
H	GP, No Cov, No Treat	22.18 [6.34]	33.71 [9.63]	15.14 [4.33]	27.37 [7.71]	10.92 [3.12]	21.40 [6.11]	10.52 [3.00]	18.42 [5.26]
I	Hibma	17.73 [5.07]	29.54 [8.44]	13.21 [3.77]	23.98 [6.85]	11.97 [3.42]	21.65 [6.19]	8.68 [2.48]	16.21 [4.63]

All the models tested appear to perform well on calibration, producing QQ plots which stay close to the target line, and thus very low SAD scores. This implies that the predicted NSAA score distributions made by the models are reasonably accurate and reliable.

The RW models consistently produce the smallest SAD scores, with each of them producing a lower score than their analogous GP model. This suggests that their structure offers better fits providing slightly more accurate results. The GP models still perform very well, with the difference between them and the RW models being fairly small. We also note that models that include the covariate terms also appear to have slightly better fits than their analogous treatment models, which suggests that including the covariate terms also improves predictive capability. No clear pattern emerges for treatments, suggesting that their inclusion does not affect the quality of the calibration.

Our models appear to generally calibrate better than the Hibma model. This is likely to be a feature of the model trajectory shape generated by the Hibma model, which flattens off at a non-zero value, rather than converging downward to zero.

With the quality of calibration examined, we can examine the size of PIs. The mean width of the 70% and 95% PIs for different numbers of training points can be seen in table 3.

Our models perform strongly here and the best appear to be able to make predictions at the 70% level to within under 3 MCIDs, and at the 95% level under 5 MCIDs, given enough points.

Similarly to the calibration exercise, the use of covariates significantly enhances the results here, with covariate models consistently producing the four smallest PIs. Furthermore, the differences between many of the covariate models and their analogous non-covariate model often amounts to a full MCID. Inclusion of treatments additionally does not provide any noticeable reduction in PI width.

The dynamics between RWs and GPs are clearly more complex in this context, showing no consistent changes in the absence of covariates. However, for models that include covariates, we see a large improvement in GP models over RW ones, which sometimes amounts to a full MCID. The result is that the best performing models here appear to be E and F: the two models with covariates and a GP.

Our models consistently provide smaller PI than the Hibma model when covariate terms are included. In combination with the much stronger accuracy of the predictions, this suggests that our covariate models perform higher quality future NSAA score prediction.

Overall, it is clear that any of the models A, B, E, F performed best at predicting NSAA scores. A and B provided the most marginally more accurate predictions; while E and F were able to make smaller PIs, which amounted to a full MCID at 95% given enough design points.

### (b) Walk time prediction

We now analyse the results from the walk time predictions. Examples of the predictions made can be seen in electronic supplementary material C, while the relevant numerical results, tables and figures for analysis can be found in electronic supplementary material G.

Overall model performance is weaker for walk times than for NSAA scores. We note that the QQ plots generally fall above the target line for all models, suggesting under-confidence.

Similarly to NSAA scores, it is notable that covariate models A, B, E, F provide better accuracy than non-covariate models C, D, G, H. The GP and covariate models, G and H, provide particularly high SAD score, leading us to conclude that their under-confidence is extreme.

We also note that each treatment model and their analogous non-treatment model have similar SAD scores and have similar curves on the QQ plot. This suggests again that the treatment term does not provide additional value as part of our modelling approach.

The inclusion of covariate models again consistently provides the smallest PIs, demonstrating the high utility of using them in these models. Models E and F, which use GPs and covariates, also almost always provide the top two smallest PIs, showing that it is clearly better to use these features when modelling walk times. Of these two, model E, which included treatments, generally performed slightly better, however the difference is small.

Models G and H provided extremely large PIs that would have very little utility in a clinical setting. This aligns with their under-confidence in calibration.

### (c) Rise from floor time prediction

Finally, we consider the performance of the RFF time predictions. Examples of such predictions can be found in electronic supplementary material D, while the relevant tables and figures for analysis can be found in electronic supplementary material H.

The models have calibrated well, with no discernible patterns observed between SAD scores and the various model versions.

Similarly to walk times, find that models E and F, which use covariates and GPs, provide the smallest PIs, alongside model B, which uses RWs. These are followed by A, which demonstrates the utility of using the covariate terms. Models G and H do not fit as poorly as in the walk times, however the broad conclusions about which models work best for RFF time are clearly the same as for walk times.

## 5. Methodology for evaluation of synthetic data quality

The secondary objective of our modelling is to generate high quality synthetic data. We measure the quality by considering how similarly distributed points in the synthetic data are to the real data, and by examining how similar the latent trajectory structures are.

To generate the synthetic data from the models, NSAA trajectories are sampled from the multivariate normal distribution used to approximate the population-level posteriors. NSAA scores must then be rounded to their nearest integer to give valid scores. We extract 294 trajectories to match the size of the validation dataset. To avoid randomness in sampled trajectories affecting the results as much as they might otherwise, we perform bootstrapping, repeating this process 100 times, taking averages of the estimated scores discussed later in the paragraph.

Before evaluating the quality of the synthetic data, we must also emulate where points are missing in the real dataset. This involves ensuring three features of each synthetic individual’s data reflect that of the real data, namely the time of their initial known point, the sampling rate (regularity of known points), and the time of their last known point. We now describe how this was done, with the process also visualized in electronic supplementary material F.

We first examine the distribution of initial points for individuals. To do this, we examine the distribution of initial points for individuals as well as the distribution of sampling rates for individuals based on the training data. This means that for each individual in the synthetic data, we sample a time that we consider the initial point, $t_{0}$ from the population distribution. Points before $t_{0}$ are then removed, making $t_{0}$ the initial point for that trajectory. We then try to make the sampling rates in the synthetic data reflect that of the real data. Similarly to how we sampled initial points from the training data, we then sample sampling rates, 1/ω from the distribution of estimated sampling rates in the training data. We then remove all points that are not in the set $\left\{t_{0}+n\omega |n\in {\mathbb{N}}_{0}\right\}$ , meaning that the remaining points are those points $t_{0}$ and those every ω time points onward from $t_{0}$ .

To determine when a trajectory stops, we partitioned the ( t , NSAA) space into a grid six boxes across and six boxes high. For each box, we use the training data to calculate the probability of a point in that box being the last point for an individual. Then some synthetic points are assigned as the last point in its trajectory, based on a Bernoulli trial for each point, with a success probability corresponding to the box to which the point belongs. If it was, all points after it in the trajectory were removed. As there were no NSAA scores of zero in the real dataset, we also removed all scores of zero in the synthetic data.

Once the generation of the synthetic data is complete, we assess how well it matches the real data. We use two measures to do this. The first is a measure of the divergence of two distributions [27]. Before doing this, both the synthetic dataset and the validation dataset must be converted into distributions, and this was done with a kernel density estimate, using the *kde2d* function in the *MASS* package in R. The Kullback–Leibler (KL) divergence between the real and synthetic datasets’ distributions is given by the following formula, as defined in [28]:


(5.1) $$\begin{array}{lcr} & KL=\sum_{i\in I}P_{v}(i)\;\log\;\frac{P_{v}(i)}{Q_{v}(i)}, & \end{array}$$

where $P_{v}$ and $Q_{v}$ are the two synthetic and real distributions, respectively, and I is the space, {1,...,34}×{1,...,69} . A score close to zero suggests the two distributions are similar to each other. We can therefore use this to check that our synthetic data points are distributed similarly to the real data points.

We must also check that the latent trajectory structure is similar. In order to do this, we hence create a measure derived from [29]. The first step in this process is to cluster individuals from the synthetic and test data together. Typically, this is done with k-means clustering; however, this approach encounters issues when dealing with a dataset with as much missing data as the one we are working with. We hence use latent cluster mixture modelling (LCMM), using a quadratic function of age, to do this, as it has been demonstrated effective for examining latent structures in NSAA scores in previous studies [8,9]. In line with these studies, we create four subgroups using the LCMM package [30]. For the synthetic data to match perfectly with the real data, we would expect the same proportion of synthetic individuals to be classified in each group as for the real validation individuals. The log-cluster (LC) measure evaluates this [28]:


(5.2) $$\begin{array}{lcr} & LC=\log\left(\frac{1}{G}\sum_{j=1}^{G}{\left(\frac{n_{j}^{R}}{n_{j}}-c\right)}^{2}\right), & \end{array}$$

where $n_{j}$ is the number of individuals in the j -th cluster, $n_{j}^{R}$ is the number of samples from the real dataset in the j -th cluster and $c=n^{R}/(n^{R}+n^{S})$ . Similarly to KL divergence, a closer value to zero indicates better performance.

## 6. Covariates improved synthetic data quality

In this section, we present the resultant LC and KL divergences based on evaluation of the synthetic data generated by the models. LC and KL divergences are given in table 4. We also present examples of synthetic data in the electronic supplementary material, section E.

**Table 4.** Table of the bootstrapped mean LC-scores achieved by LCMM clustering of the synthetic data generated by the models, and the bootstrapped mean KL divergences generated. Lower LC-scores suggest similar latent structures between the synthetic data and the real data, while KL divergences closer to zero suggest that the synthetic data and the real data cover similar spaces.

label	model description	LC score	LC rank	KL divergence	KL rank
A	RW, Cov, Treat	−6.84	1	0.138	1
B	RW, Cov, No Treat	−6.23	3	0.165	4
C	RW, No Cov, Treat	−3.53	8	0.423	8
D	RW, No Cov, No Treat	−3.49	9	0.444	9
E	GP, Cov, Treat	−6.35	2	0.147	2
F	GP, Cov, No Treat	−6.18	4	0.159	3
G	GP, No Cov, Treat	−3.70	7	0.416	7
H	GP, No Cov, No Treat	−3.75	6	0.408	6
I	Hibma	−5.28	5	0.245	5

In this synthetic data generation task, we see a similar pattern emerging as we found in the prediction tasks: models that utilize covariates perform much better than those that do not. It is interesting to note that the models including treatment terms generally provide better synthetic results than their analogous non-treatment models, as this contrasts to the results from the prediction tasks, in which inclusion of treatment terms made little difference. This effect is, however, small. The GP models also do not perform much better than the RW models.

The Hibma model, I, performs in the middle of the group, yielding better results than the non-covariate models but worse than the covariate models.

Model A, which includes RWs, covariates and treatments performs the best in both tasks, suggesting that it could be the best model to use for stratification. We note that the differences between model A and the other covariate models may not be significant.

## 7. Discussion

In this paper, we develop a new model for predicting NSAA scores, walk times and rise from floor times in DMD patients. Our objectives are twofold: to improve probabilistic predictions of these outcomes at an individual level and to generate new synthetic datasets. To evaluate the utility of incorporating a GP or a random walk structure, covariates between parameters for each outcome, and a treatment term, we created eight model variations for comparison. These were also compared with a previously existing NSAA modelling framework. We assess the quality of predictions by examining the quantile coverage of test points within the PIs and the narrowness of these PIs. We also use the models to generate synthetic data, the quality of which is evaluated using KL scores to measure the similarity of point distributions between synthetic and real data, and LC scores derived from LCMM clustering to compare the latent structures of the two datasets. We show that our models performed very well at accurately predicting NSAA scores, with all models achieving small sums of the absolute differences scores for the outcome and QQ plots that kept closely to the target line. This provides strong evidence for the validity of the use of the models for NSAA score prediction.

To the best of our knowledge the Hibma model [13] represents the most similar attempt to ours for modelling NSAA score trajectories to date. We have shown here that model F, which included a GP, covariates between outcomes, and no treatment term, performed strongly across all tasks and consistently outperformed Hibma. It sometimes did so by as much as a full minimum clinically relevant difference and had improved calibration. When sufficiently many training points were available, it achieved PI widths of under 8.19 NSAA (2.34 MCIDs) for 70% PIs and 15.38 NSAA (4.12 MCIDs) for 95% PIs, while achieving higher predictive accuracy with a SAD score of 2.39. The model was also highly effective at predicting walk times and RFF times. Our modelling framework thus represents a clear improvement over currently available approaches. Models E and F performed similarly across all prediction tasks, however, with the objective of keeping the models simple and number of parameters low, it could be argued that F would be better to use in practice.

Another noteworthy observation from our investigation is the strength of models that utilize covariate terms over those that do not. Covariate models provided substantially better results over almost all the tasks, which would support the approach of using co-predictors with NSAA scores in a similar manner in future models.

We also observed an advantage to using treatment terms in the synthetic data tasks. This could be important if the model is used for augmentation because the synthetic data would be able to account for treatment effects. Using treatment terms on the prediction task, on the other hand, did not appear to provide a consistent advantage. This is an interesting result, which could, superficially, suggest that the treatments are having little to no effect on the trajectories. This is unlikely, as the positive effects of the steroid treatments are well established. A likelier cause is that those patients that did not receive treatments are not a control for those who did, and this creates a non-identifiability issue between the treatment effects, Γ , and the disease progression rate, Δ . Additionally, treatments were generally started towards the top of the plateau in NSAA score, at the same time the *softplus* on the disease effect begins to activate in our model. This means that the treatment effect size could not be determined from a spontaneous improvement or slowing of deterioration when the treatment starts. These issues likely resulted in minimal improvements to the model from the term’s inclusion.

### (a) Limitations

There are certain limitations to the methodology we have used. Firstly, we do not model where data are missing, instead we implement an algorithm that imperfectly removes data to reflect where points are present in the real data as much as possible. This means that the KL divergences, and to a lesser extent, the log-cluster scores, do not necessarily provide a full depiction of how effective the models can be and should be primarily used only for comparison of synthetic data that have gone through the same data removal process (i.e. not be compared with models from outside this study based on different modelling approaches). This is not a major concern with regard to the quality of the synthetic data, as full datasets would typically be more useful for research than ones with large amounts of missing data.

A second limitation relates to the efficacy of performing a model selection task at the same time as testing the model. When testing multiple models, it is inevitable that some models will perform better than others. By selecting the most effective one and presenting that as the most powerful version, we risk an effect, where the best performing model may not do as much better than the others as it may appear here.

Another point to note is our comparison with the Hibma model. In its original form, the Hibma model is presented alongside a loss of ambulation model and is not intended for future score prediction. This means that its characteristic of flattening off at a non-zero value, rather than converging downward towards zero, was less significant in that paper than it is for our purposes. Thus, it could be argued that our use of the Hibma model here is not entirely analogous. However, our model, which shows NSAA scores converging to zero with continued ambulation, appears to fit the data more accurately.

Our model produced PIs that are narrower than the model we use for comparison, while still allowing for a reasonable degree of variability. However, over a longer time horizon, this level of uncertainty may hinder meaningful insights for clinicians and families. That said, it is challenging to determine how much further these intervals could be tightened through improved modelling, as a significant portion of the remaining uncertainty may be inherent and aleatoric in nature.

Our model includes an assumption in its choice of the correlation length of the selected square exponential kernel. One limitation of this approach is that is not able to capture higher frequencies in NSAA scores when making predictions or when generating synthetic data.

We did not conduct a rigorous assessment of computational efficiency during model training. However, we observed that differences in MCMC fitting times among the models were minimal, with all requiring approximately 1 week to train. It is worth noting that the RW models exhibited significantly lower memory usage, which could make them more suitable for training on lower performance computers. Additionally, this lower memory requirement might enable fitting multiple MCMC chains in parallel rather than sequentially, potentially reducing the overall training time considerably.

### (b) Future work

One feature of the models demonstrated in this paper is that they include few mechanisms or parameters that relate specifically to DMD. This means that there is potential for this modelling approach to be used for individual-level prediction of clinical outcomes associated with other degenerative diseases. This is currently untested, but there is high potential for future work to explore this possibility.

We focus on modelling scores, with the whereabouts of missing points of data being largely outside the scope of our investigation. This has some implications for the synthetic data generation pipeline, in which we use relatively simple techniques based on sampling regularities and stopping times using a grid system. However, other opportunities exist, for example [13], originally presents the base-NSAA model (model I in our paper) with a survival model to predict loss of ambulation. The potential to integrate their procedures or experiments with our models may arise in the future.

An option that could be worth exploring in the future could be creating predictions through averaging trajectories from several models, taking an ensemble. There would be limited utility in doing this over our models A–H, as they create broadly similar shapes. However, averaging ours with the Hibma model and other models that may be developed in the future could potentially provide a highly effective prediction.

### (c) Concluding remarks

In this paper, we presented various iterations of our dynamical linear model, which were validated by assessing the accuracy and width of the generated PIs, as well as the quality of the synthetic data produced. Models E and F, which incorporated a GP structure and covariates, demonstrated high effectiveness in predicting future NSAA scores, walk times and RFF times in DMD patients. Model A, which is based on random walks, however, appeared strongest on the synthetic data generation tasks. Our models also showed improvements over a previously proposed, less personalized modelling approach.

## Data Availability

The code is available via [31]. Supplementary material is available online [32].

## References

[1] Crisafulli S , Sultana J , Fontana A , Salvo F , Messina S , Trifirò G . 2020 Global epidemiology of Duchenne muscular dystrophy: an updated systematic review and meta-analysis. Orphanet J. Rare Dis **15** , 141. (10.1186/s13023-020-01430-8) 32503598 PMC7275323

[2] Gao QQ , McNally EM . 2015 The dystrophin complex: structure, function, and implications for therapy. In Compr. physiol (ed. R Terjung ), pp. 1223–1239, vol. **5** , 1st edn. Wiley. (10.1002/cphy.c140048) PMC4767260 26140716

[3] Broomfield J , Hill M , Guglieri M , Crowther M , Abrams K . 2021 Life expectancy in duchenne muscular dystrophy. Neurology (ECronicon). **97** , 2304–2314. (10.1212/WNL.0000000000012910) PMC8665435 34645707

[4] Naarding KJ *et al* . 2023 269th ENMC international workshop: 10 years of clinical trials in Duchenne muscular dystrophy - What have we learned? 9-11 December 2022, Hoofddorp, The Netherlands. Neuromuscul. Disord. **33** , 897–910. (10.1016/j.nmd.2023.10.003) 37926638

[5] Fischer R , Porter K , Donovan JM , Scavina MT , Armstrong N , Denger B , Hasham S , Peay H . 2023 A mixed-method study exploring patient-experienced and caregiver-reported benefits and side effects of corticosteroid use in Duchenne Muscular Dystrophy. J. Neuromuscul. Dis. **10** , 593–613. (10.3233/JND-221617) 37182893 PMC10357210

[6] Giuffrè M , Shung DL . 2023 Harnessing the power of synthetic data in healthcare: innovation, application, and privacy. NPJ Digit. Med. **6** , 186. (10.1038/s41746-023-00927-3) 37813960 PMC10562365

[7] Hahn W , Schütte K , Schultz K , Wolkenhauer O , Sedlmayr M , Schuler U , Eichler M , Bej S , Wolfien M . 2022 Contribution of synthetic data generation towards an improved patient stratification in palliative care. J. Pers. Med. **12** , 1278. (10.3390/jpm12081278) 36013227 PMC9409663

[8] Muntoni F , Domingos J , Manzur AY , Mayhew A , Guglieri M , Sajeev G , Signorovitch J , Ward SJ , UK NorthStar Network . 2019 Categorising trajectories and individual item changes of the North Star Ambulatory Assessment in patients with Duchenne muscular dystrophy. PLoS One (ed. D Ribeiro ), **14** , e0221097. (10.1371/journal.pone.0221097) 31479456 PMC6719875

[9] Fang Y , McDonald CM , Clemens PR , Gordish HD , Illei K , Hoffman EP , Dang UJ , CINRG DNHS and Vamorolone 002/003/LTE Investigators . 2023 Modeling early heterogeneous rates of progression in boys with Duchenne Muscular Dystrophy. J. Neuromuscul. Dis. **10** , 349–364. (10.3233/JND-221527) 36806514 PMC10200136

[10] Rooney WD *et al* . 2020 Modeling disease trajectory in Duchenne muscular dystrophy. Neurology **94** , e1622–e1633. (10.1212/WNL.0000000000009244) 32184340 PMC7251517

[11] Hamuro L , Chan P , Tirucherai G , AbuTarif M . 2017 Developing a natural history progression model for Duchenne muscular dystrophy using the six-minute walk test. CPT. Pharmacometrics Syst. Pharmacol. **6** , 596–603. (10.1002/psp4.12220) 28643370 PMC5613187

[12] Lennie JL , Mondick JT , Gastonguay MR . 2020 Latent process model of the 6-minute walk test in Duchenne muscular dystrophy: a Bayesian approach to quantifying rare disease progression. J. Pharmacokinet. Pharmacodyn. **47** , 91–104. (10.1007/s10928-020-09671-7) 31960231

[13] Hibma JE , Jayachandran P , Neelakantan S , Harnisch LO . 2023 Disease progression modeling of the North star ambulatory assessment for Duchenne muscular dystrophy. CPT. Pharmacometrics Syst. Pharmacol. **12** , 375–386. (10.1002/psp4.12921) 36718719 PMC10014057

[14] Guglieri M *et al* . 2022 Efficacy and safety of vamorolone vs placebo and prednisone among boys with Duchenne muscular dystrophy: a randomized clinical trial. JAMA Neurol. **79** , 1005–1014. (10.1001/jamaneurol.2022.2480) 36036925 PMC9425287

[15] Peng F , Xu H , Song Y , Xu K , Li S , Cai X , Guo Y , Gong L . 2023 Longitudinal study of multi-parameter quantitative magnetic resonance imaging in Duchenne muscular dystrophy: hyperresponsiveness of gluteus maximus and detection of subclinical disease progression in functionally stable patients. J. Neurol. **270** , 1439–1451. (10.1007/s00415-022-11470-8) 36385201

[16] Kenis-Coskun O , Imamoglu S , Karamancioglu B , Kurt K , Ozturk G , Karadag-Saygi E . 2022 Comparison of telerehabilitation versus home-based video exercise in patients with Duchenne muscular dystrophy: a single-blind randomized study. Acta Neurol. Belg. **122** , 1269–1280. (10.1007/s13760-022-01975-4) 35616780 PMC9133319

[17] Flanigan KM *et al* . 2022 A first-in-human phase I/IIa gene transfer clinical trial for Duchenne muscular dystrophy using rAAVrh74.MCK.GALGT2. Mol. Ther. Methods Clin. Dev. **27** , 47–60. (10.1016/j.omtm.2022.08.009) 36186954 PMC9483573

[18] Sherlock SP *et al* . 2022 Quantitative magnetic resonance imaging measures as biomarkers of disease progression in boys with Duchenne muscular dystrophy: a phase 2 trial of domagrozumab. J. Neurol. **269** , 4421–4435. (10.1007/s00415-022-11084-0) 35396602 PMC9294028

[19] R.Core Team . 2021 R: a language and environment for statistical computing. Vienna, Austria: R Foundation for Statistical Computing.

[20] de Valpine P , Turek D , Paciorek CJ , Anderson-Bergman C , Lang DT , Bodik R . 2017 Programming With models: writing statistical algorithms for general model structures with NIMBLE. J. Comput. Graph. Stat. **26** , 403–413. (10.1080/10618600.2016.1172487)

[21] NIMBLE Development team . 2023 NIMBLE: MCMC, particle filtering, and programmable hierarchical modeling (10.5281/ZENODO.8047617)

[22] Gramacy RB . 2020 Surrogates: Gaussian process modeling, design and optimization for the applied sciences. texts in statistical science. Boca Raton, FL: Chapman Hall/CRC. (10.1201/9780367815493)

[23] Roberts S , Osborne M , Ebden M , Reece S , Gibson N , Aigrain S . 2013 Gaussian processes for time-series modelling. Philos. Trans. R. Soc. **371** , 20110550. (10.1098/rsta.2011.0550) 23277607

[24] Eaton ML . 2007 Multivariate statistics: a vector space approach. vol. 53. Beachwood, OH: Institute of Mathematical Statistics.(Lecture Notes-Monograph Series). (10.1214/lnms/1196285102)

[25] Copay AG , Subach BR , Glassman SD , Polly DW , Schuler TC . 2007 Understanding the minimum clinically important difference: a review of concepts and methods. Spine J. **7** , 541–546. (10.1016/j.spinee.2007.01.008) 17448732

[26] Ayyar Gupta V *et al* . 2023 Determining minimal clinically important differences in the North Star Ambulatory Assessment (NSAA) for patients with Duchenne muscular dystrophy. PLoS One **18** , e0283669. (10.1371/journal.pone.0283669) 37099511 PMC10132589

[27] Kullback S , Leibler RA . 1951 On information and sufficiency. Ann. Math. Stat. **22** , 79–86. (10.1214/aoms/1177729694)

[28] Goncalves A , Ray P , Soper B , Stevens J , Coyle L , Sales AP . 2020 Generation and evaluation of synthetic patient data. BMC Med. Res. Methodol. **20** , 108. (10.1186/s12874-020-00977-1) 32381039 PMC7204018

[29] Woo MJ , Reiter JP , Oganian A , Karr AF . 2009 Global measures of data utility for microdata masked for disclosure limitation. J. Priv. Confidentiality **1** . (10.29012/jpc.v1i1.568)

[30] Proust-Lima C , Philipps V , Liquet B . 2017 Estimation of extended mixed models using latent classes and latent processes: the R package **lcmm** . J. Stat. Softw. **78** , 02. (10.18637/jss.v078.i02)

[31] Applebaum V . 2024 VictorApplebaum/FullyPersonalisedDMDModel: fully personalised modelling of Duchenne Muscular Dystrophy ambulation (1.0). Zenodo. (10.5281/zenodo.14538377) PMC12105785 40172561

[32] Applebaum V , Baker E , Kim T , Stimpson G , Challenor P , Wedgwood KCA *et al* . 2025 Supplementary material from: Fully personalised modelling of Duchenne Muscular Dystrophy ambulation. Figshare. (10.6084/m9.figshare.c.7715262) PMC12105785 40172561

